# Acute inflammation reveals GABA_A_ receptor‐mediated nociception in mouse dorsal root ganglion neurons via PGE_2_ receptor 4 signaling

**DOI:** 10.14814/phy2.13178

**Published:** 2017-04-24

**Authors:** In Jeong Jang, Alexander J. Davies, Nozomi Akimoto, Seung Keun Back, Pa Reum Lee, Heung Sik Na, Hidemasa Furue, Sung Jun Jung, Yong Ho Kim, Seog Bae Oh

**Affiliations:** ^1^ Pain LaboratoryDental Research Institute and Department of Neurobiology and PhysiologySchool of DentistrySeoul National UniversitySeoulKorea; ^2^ Department of Brain and Cognitive SciencesCollege of Natural SciencesSeoul National UniversitySeoulKorea; ^3^ Department of Information PhysiologyNational Institute for Physiological SciencesOkazakiJapan; ^4^ Department of PhysiologyKorea University College of MedicineSeoulKorea; ^5^ Department of Pharmacology and BiotechnologyCollege of Medical EngineeringKonyang UniversityDaejeonKorea; ^6^ Department of PhysiologyHanyang UniversitySeoulKorea

**Keywords:** EP4 receptors, formalin test, Gamma‐aminobutyric acid, peripheral sensitization, prostaglandin E2, TTX‐resistant sodium channels

## Abstract

Gamma‐aminobutyric acid (GABA) depolarizes dorsal root ganglia (DRG) primary afferent neurons through activation of Cl^−^ permeable GABA_A_ receptors but the physiologic role of GABA_A_ receptors in the peripheral terminals of DRG neurons remains unclear. In this study, we investigated the role of peripheral GABA_A_ receptors in nociception using a mouse model of acute inflammation. In vivo, peripheral administration of the selective GABA_A_ receptor agonist muscimol evoked spontaneous licking behavior, as well as spinal wide dynamic range (WDR) neuron firing, after pre‐conditioning with formalin but had no effect in saline‐treated mice. GABA_A_ receptor‐mediated pain behavior after acute formalin treatment was abolished by the GABA_A_ receptor blocker picrotoxin and cyclooxygenase inhibitor indomethacin. In addition, treatment with prostaglandin E2 (PGE_2_) was sufficient to reveal muscimol‐induced licking behavior. In vitro, GABA induced sub‐threshold depolarization in DRG neurons through GABA_A_ receptor activation. Both formalin and PGE_2_ potentiated GABA‐induced Ca^2+^ transients and membrane depolarization in capsaicin‐sensitive nociceptive DRG neurons; these effects were blocked by the prostaglandin E2 receptor 4 (EP4) antagonist AH23848 (10 *μ*mol/L). Furthermore, potentiation of GABA responses by PGE_2_ was prevented by the selective Na_v_1.8 antagonist A887826 (100 nmol/L). Although the function of the Na^+^‐K^+^‐2Cl^‐^ co‐transporter NKCC1 was required to maintain the Cl^‐^ ion gradient in isolated DRG neurons, NKCC1 was not required for GABA_A_ receptor‐mediated nociceptive behavior after acute inflammation. Taken together, these results demonstrate that GABA_A_ receptors may contribute to the excitation of peripheral sensory neurons in inflammation through a combined effect involving PGE_2_‐EP4 signaling and Na^+^ channel sensitization.

## Introduction

Doral root ganglion (DRG) neurons are primary afferent neurons which conduct sensory information from the environment to the spinal cord. DRG neurons express the Cl^‐^ permeable gamma‐aminobutyric acid (GABA)_A_ receptor (Morris et al. 1983; Farrant and Nusser 2005; Zeilhofer et al. 2012) which display membrane depolarization in response to GABA stimulation. It is suggested that GABA_A_ receptor‐mediated tonic primary afferent depolarization (PAD) may serve to inhibit primary afferent signaling through the inactivation of voltage‐gated channels such as Na_v_ and thus reduce neurotransmitter release from the afferent terminals to second‐order neurons (Willis 1999; Kullmann et al. 2005; Guo and Hu 2014). In addition, GABA may itself exert an inhibitory effect via activation of metabotropic GABA_B_ receptors expressed on DRG neurons (Hanack et al. 2015).

It has previously been demonstrated that co‐application of a low dose (2 *μ*mol/L, 30 *μ*L) of the GABA_A_ receptor agonist muscimol with formalin into the mouse hind paw reduced formalin‐induced biphasic nocifensive behavior (Carlton et al. 1999), a result consistent with the inhibition associated with PAD. However, a high dose of muscimol (1 mmol/L) was in fact found to *increase* formalin‐induced biphasic nocifensive behavior (Carlton et al. 1999; Bravo‐Hernandez et al. 2014), a phenomenon that was blocked by pre‐treatment with the GABA_A_ receptor antagonist bicuculline (Bravo‐Hernandez et al. 2014). These findings suggest that near‐maximally activated GABA_A_ receptors may participate in nociceptive sensory transduction in pathological conditions. However, the molecular mechanism(s) underlying the contribution of peripheral GABA_A_ receptors to inflammatory pain remain unclear.

In the present study, we sought to investigate the mechanism behind this apparent shift in the role of peripheral GABA_A_ receptors in acute inflammation. We found that peripheral GABA_A_ receptor activation induces de novo pain behavior after formalin and prostaglandin E2 (PGE_2_) pre‐conditioning through a signaling pathway involving EP4 receptor activation. We also found that functional upregulation of tetrodotoxin‐resistant voltage‐gated sodium channels in the presence of PGE_2_ allows GABA signaling to evoke robust neuronal activity under inflammatory conditions. These findings may open new avenues of research into the contribution of GABA_A_ receptors to sensory physiology.

## Materials and Methods

### Ethical approval

All surgical and experimental procedures were reviewed and approved by the Institutional Animal Care and Use Committees of Seoul National University (SNU‐120710‐5‐1) and the National Institute of Physiological Sciences (NIPS), Japan. Experiments were carried out and are reported here in accordance with the ARRIVE guidelines (Kilkenny et al. 2010) and the principles and best practice of The Journal of Physiology (Grundy 2015).

### Animals

Adult C57BL/6J (wild type) male mice (6–8 weeks) were purchased from Daehan Biolink (Korea) and Japan SLC (Hamamatsu, Japan). Adult male and female Na^+^‐K^+^‐2Cl^−^ co‐transporter 1 knockout (NKCC1^−/−^) mice were from a stock originally created by Dr. Gary Shull of the University of Cincinnati (Flagella et al. 1999) and maintained at the Department of Physiology, Korea University, Seoul. Animals were housed four to five mice per cage in a conventional facility with a 12 h light cycle (lights on 8.00 am) and ad libitum access to water and chow. Mice were acclimatized for at least 1 week before experiments. All behavioral experiments were carried out between 12.00 pm and 6.00 pm. Drug treatments were assigned randomly to mice of the same litter by an independent observer. Mice were euthanized at the end of behavior experiments by rising concentration of CO_2_, or by overdose of urethane followed by cervical dislocation at the end of in vivo recording, in accordance with the Schedule 1 of the UK Home Office Animals (Scientific Procedures) Act 1986.

### Formalin pre‐conditioning and spontaneous pain behavior

Mice were placed in an observation chamber (60 × 100 × 60 mm) and allowed to habituate for at least 30 min before drug administration. A mirror was positioned behind the observation chamber to provide an unobstructed view. After habituation, one experimenter restrained the mouse while another experimenter performed a ‘pre‐conditioning’ injection of formalin (0.8%) or saline vehicle (20 *μ*L) subcutaneously into the dorsum of the right hind paw using a 0.3 mL insulin syringe fitted with a 31 gauge needle. After subsidence of formalin‐induced nocifensive behaviors (70 min after first injection) a second ‘stimulating’ injection of muscimol (1 mmol/L) or saline vehicle (20 *μ*L) was given subcutaneously to the same dorsum area of the hind paw. For inhibition of GABA_A_ receptors in vivo, picrotoxin was added at the indicated concentration to both the initial formalin solution and muscimol stimulation solution before paw injection. Care was taken with both injections to avoid leakage of the solutions from the paw. Spontaneous pain behaviors were assessed by measuring the time each animal spent licking the affected hind paw. The cumulative time spent licking was recorded during the 5 min immediately before drug administration and up to 110 min after the first drug administration. All behavior tests were video recorded and analyzed offline by an investigator who was blind to the treatment and genetic background of the mice.

### In vivo spinal cord extracellular recording

The methods used for the present study were modifications of those used in preceding studies (Sugiyama et al. 2012; Funai et al. 2014). Adult C57BL/6J male mice were anesthetized with urethane (1.5 g/kg, i.p.) and monitored for loss of hind paw pinch reflex with supplemental injections of urethane (0.2 g/kg, i.p.) given if necessary during the experiment. A laminectomy was performed to expose the lumbar enlargement of the spinal cord. The mouse was placed in a stereotaxic apparatus (Model STS‐A, Narishige, Tokyo, Japan). After the dura mater was opened, the pia‐arachnoid membrane was cut to make a window to allow a tungsten electrode to enter into the spinal cord. The surface of the spinal cord was irrigated with 95% O_2_ and 5% CO_2_ equilibrated Krebs solution (in mmol/L: 117 NaCl, 3.6 KCl, 2.5 CaCl_2_, 1.2 MgCl_2_, 1.2 NaH_2_PO_4_, 11 glucose, and 25 NaHCO_3_) at a flow rate of 10–15 mL/min at 38°C ± 1°C. The tungsten electrode (impedance, 1 MX, A‐M systems, Sequim, WA) was advanced into the spinal cord using a micromanipulator (Model SM‐11, Narishige). The tungsten electrode was placed into the spinal cord dorsal horn and action potentials in spinal cord neurons were extracellularly recorded with an AC differential amplifier (DAM 80, World Precision Instruments, Sarasota, FL). Wide dynamic range (WDR) neurons with a receptive field covering the ipsilateral hind paw were identified during electrode advancement by consistent spike responses to touch, brush and pinch stimuli applied to the hind paw. The firing rate of spinal cord neurons was analyzed with Offline Sorter software (version 3, Plexon, Dallas, TX). Injections were made into the dorsal surface of the ipsilateral hind paw in the same manner as for the behavioral experiments.

### DRG preparation

DRG neurons were isolated from 6 to 8‐week‐old mice. Animals were killed in accordance with the Schedule 1 of the UK Home Office Animals (Scientific Procedures) Act 1986 by inhalation of a rising, lethal concentration of isoflurane (Hana Pharm. Co. Ltd., Korea) followed by decapitation. Bilateral DRG were rapidly removed under aseptic conditions and placed in ice‐cold HBSS (Gibco) containing 20 mM HEPES. DRGs were digested in 1 mg/ml collagenase A (Roche) and 2.4 U/ml dispase II (Roche) in HBSS for 60 min, respectively, followed by 5 min in 0.25% trypsin (Sigma), all at 37°C. The DRGs were then washed in DMEM (Gibco) and resuspended in DMEM medium supplemented with 10% FBS (Invitrogen) and 1% penicillin/streptomycin (Sigma). DRGs were then mechanically dissociated using fire‐polished glass pipettes, centrifuged (200 g, 5 min, resuspended in Neurobasal media (Gibco) with B27 supplement (Invitrogen), L‐glutamine and 1% penicillin/streptomycin (Invitrogen), and plated on 0.5 mg/ml poly‐D‐lysine (Sigma)‐coated glass coverslips. Cells were maintained at 37°C in a 5% CO_2_ incubator. All experiments using DRG neurons were performed 12–36 h after plating.

### Patch‐clamp electrophysiology

Electrophysiologic responses were recorded using the patch‐clamp recording technique with EPC‐10 amplifier and Pulse 8.30 software (both from HEKA). For perforated‐patch recordings in DRG neurons, we used an external bath solution of the following composition (in mmol/L): 140 NaCl, 5 KCl, 2 CaCl_2_, 1 MgCl_2_, 10 glucose, and 10 HEPES, adjusted to pH 7.4 with NaOH. Patch pipettes with resistances of 3–5 MΩ were made from borosilicate glass capillaries. Pipette solution contained (in mM): 140 K‐gluconate, 1 CaCl_2_, 2 MgCl_2_, 10 EGTA, 5 K_2_ATP, 10 HEPES, adjusted to pH 7.4 with KOH. A 50 mg/mL stock solution of gramicidin (Calbiochem, La Jolla, CA) was prepared in dimethylsulfoxide (DMSO; Sigma). Gramicidin was diluted into the pipette solution to a final concentration of 100 *μ*g/mL and vortexed thoroughly before use. All drug solutions were applied to cells by local perfusion through a capillary tube (1.1 mm inner diameter) positioned near the cell of interest. After the formation of a tight seal, the progress of gramicidin perforation was evaluated by monitoring the capacitive current transient produced by a 10 msec hyperpolarizing voltage step (−5 mV) from a holding potential of −60 mV. Cells were accepted for recording if the access resistance dropped to 20 MΩ within 20 min after seal formation. The solution flow was driven by gravity (flow rate, 3–5 mL/min) and controlled by miniature solenoid valves (The Lee Company). Perforated‐patch recordings of membrane potential (*V*_m_) were corrected offline using the following formula: *V*_m_ = *V*_p_+*V*_pf_‐(LJP), where *Vp* is the recorded potential, *V*_pf_ is the perforated‐patch potential and LJP is the liquid junction potential between intracellular pipette and extracellular bath solutions. *V*_pf_ (+3.6 mV) was measured directly as the difference in membrane potential between perforated and whole‐cell patch configuration; LJP (+16.1 mV) was calculated using JPCalc for Windows (Barry 1994) (Molecular Devices). For whole‐cell patch‐clamp recordings of sodium currents the pipette solution contained (in mmol/L): 130 CsCl, 9 NaCl, 1 MgCl_2_, 10 EGTA, 10 HEPES, adjusted to pH 7.4 with CsOH. The external solution was composed of (in mmol/L): 131 NaCl, 10 TEACl, 10 CsCl, 1 CaCl_2_, 2 MgCl_2_, 0.1 CdCl_2_, 3 4‐aminopyridine, 10 HEPES, 10 glucose adjusted to pH 7.4 with NaOH. Tetrodotoxin (TTX)‐resistant currents were recorded from DRG neurons in the presence of TTX (300 nmol/L). For quantification of current amplitude in the presence of PGE_2_ currents were evoked with a voltage pulse to 0 mV from a holding potential of −70 mV. To determine the voltage of activation of Na_v_1.8‐mediated sodium currents, a 500‐msec pre‐pulse step at −50 mV, designed to inactivate TTX‐resistant Na_v_1.9 channels (Berta et al. 2008), was followed by a series of voltage steps increments of +5 mV at a frequency of 1 Hz. A liquid junction potential of +6.0 mV was corrected offline for calculation of the conductance‐voltage relationship. Data were fit with a Boltzmann function. The shift in voltage dependence of Na_v_1.8 activation was calculated from the difference in V_1/2_ activation.

### Ca^2+^ imaging

We performed fura‐2 AM‐based (Molecular Probes) Ca^2+^ imaging experiments. Briefly, DRG neurons prepared as above were loaded with fura‐2 AM (2 *μ*mol/L) in DMEM for 50 min at 37°C in a 5% CO_2_ incubator. The cells were then rinsed with DMEM and incubated for an additional 20 min to de‐esterify the dye. Cells on slides were placed onto an inverted microscope and illuminated with a 175 W xenon arc lamp; excitation wavelengths (340/380 nm) were selected by a monochromatic wavelength changer. Intracellular calcium concentrations ([Ca^2+^]_i_) were measured by digital video microfluorometry with an intensified charge‐coupled‐device camera (CasCade, Roper Scientific) coupled to the microscope and a computer with Metafluor software (Universal Imaging). All drugs were applied via bath perfusion at a flow rate of 3–5 mL/min. For Cl^‐^ gradient depletion experiments, NaCl was substituted for equimolar Na‐gluconate in the bath solution. The left axis of all scale bars for Ca^2+^ imaging traces represents the F_340/380_ ratio.

### Drugs

Capsaicin, formalin, prostaglandin E2, thapsigargin, picrotoxin, lidocaine, *γ*‐aminobutyric acid (GABA), muscimol (3‐hydroxy‐5‐aminomethyl‐isoxazole), HC‐030031 (1,2,3,6‐tetrahydro‐1,3‐dimethyl‐N‐ [4‐ (1‐methylethyl) phenyl] ‐ 2,6‐dioxo‐7H‐purine‐7‐acetamide), bumetanide (3‐(aminosulfonyl)‐5‐(butylamino)‐4‐phenoxy benzoic acid), AH23848 ((4Z)‐7‐[(rel‐1S,2S,5R)‐5‐((1,1′‐biphenyl‐4‐yl)methoxy)‐2‐(4‐morpholinyl)‐3‐oxocyclopentyl]‐4‐heptenoic acid hemicalcium salt), AH6809 (6‐isopropoxy‐9‐oxoxanthene‐2‐carboxylic acid) and A887826 (5‐(4‐butoxy‐3‐chlorophenyl)‐N‐[[2‐(4‐morpholinyl)‐3‐pyridinyl]methyl]‐3‐pyridinecarboxamide) were purchased from Sigma. For in vivo experiments, formalin and muscimol were dissolved in 0.9% saline with sonication. For in vivo experiments, AH6809, A887826, bumetanide, and capsaicin were dissolved in 100% DMSO as stocks at 10 mmol/L and AH23848 was dissolved in saline +2% DMSO; all drugs were further diluted in bath solution immediately before experiments.

### Statistical analysis

Data are expressed as mean ± standard error of the mean (SEM) unless otherwise indicated. In in vitro experiments, ‘*n*’ refers to the number of cells recorded; in Ca^2+^ imaging experiments, the number of coverslips and number of mice used (i.e. repeat experiments) are also indicated. In behavior testing experiments, ‘*n*’ indicates the number of animals tested. Sample sizes were based on the previous literature. Statistical analyses of behavioral data were performed with two‐way ANOVA followed by Bonferroni post‐test. For all other studies, results were analyzed using one‐way ANOVA, Student's *t* test and Wilcoxon signed‐rank test as indicated. All statistical tests were performed with GraphPad Prism (version 5.00 for Windows, GraphPad Software, San Diego, California, USA); *P *<* *0.05 was considered statistically significant.

## Results

### GABA induces Ca^2+^ transients by GABA_A_ receptor activation in DRG neurons

**Figure 1:**
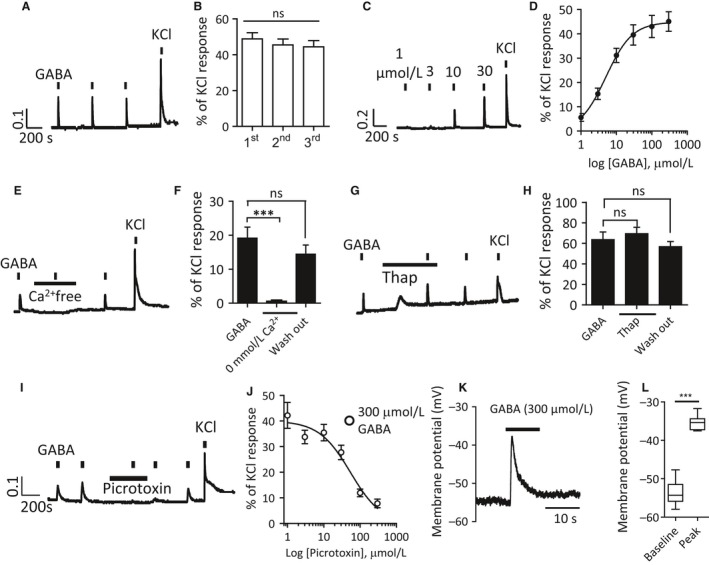
GABA induces Ca^2+^ transients via membrane depolarization in mouse dorsal root ganglion neurons. (A) Sequential application of GABA (300 *μ*mol/L, 10 sec) induces Ca^2+^ transients in DRG neurons. (B) Mean relative amplitude of sequential GABA‐induced Ca^2+^ responses (*n* = 54 neurons, 6 coverslips from 4 mice; ns, not significant, one‐way ANOVA). (C) Ca^2+^ transients are elicited by GABA in a concentration‐dependent manner. (D) Concentration‐response curve of GABA in cultured DRG neurons (*n* = 22–32 neurons, 9 coverslips from 4 mice). (E) GABA‐induced Ca^2+^ responses are abolished by extracellular Ca^2+^‐free solution. (F) Normalized Ca^2+^ responses relative to peak amplitude of 50 mmol/L KCl response (*n* = 16 cells, coverslips from 3 mice; ***P <* *0.001, one‐way ANOVA with Bonferroni post‐test). (G) Thapsigargin (1 *μ*mol/L) treatment to deplete intracellular Ca^2+^ stores had no effect on GABA‐induced Ca^2+^ transients (H) Quantification of normalized Ca^2+^ responses relative to peak amplitude of 50 mmol/L KCl response (*n* = 21 cells, 3 coverslips from 3 mice; one‐way ANOVA with Bonferroni post‐test). (I) Ca^2+^ transients evoked by supramaximal GABA (300 *μ*mol/L, 10 sec) are blocked by picrotoxin (300 *μ*mol/L). (J) Concentration‐response curve of GABA (300 *μ*mol/L)‐induced Ca^2+^ responses inhibited by picrotoxin. (K) Gramicidin perforated patch‐clamp recording of adult mouse DRG neurons. Supramaximal GABA (300 *μ*mol/L) application led to a fast, rapidly decaying depolarization of the membrane potential in small‐sized (12–20 *μ*m diameter) DRG neurons. (L) Box and whisker plot showing the mean membrane potential at rest (−53.7 ± 0.8 mV) and peak (−35.3 ± 0.6 mV) during GABA (300 *μ*mol/L) application (***P <* *0.001, paired *t*‐test; *n* = 12 neurons, 12 coverslips from 4 mice). Whiskers above and below represent max and min values, respectively. DRG, dorsal root ganglia.

We first employed ratiometric Ca^2+^ imaging to study the physiologic role of GABA_A_ receptors in acutely dissociated adult mouse DRG neurons without disrupting the intracellular ionic milieu. GABA (300 *μ*mol/L, 10 sec) applied via the bath perfusion induced Ca^2+^ transients (Fig. 1A) in a subpopulation of DRG neurons (52.8%; *n* = 507/960) (Fig. 4D). These transients were reproducible and stable across sequential applications of GABA (Fig. 1B). The amplitude of GABA‐induced Ca^2+^ transients was concentration‐dependent (Fig. 1C, D) with 300 *μ*mol/L GABA representing a supramaximal response in our Ca^2+^ assay (Fig. 1D).

The GABA_A_ receptor selectively conducts anions through its pore; therefore, we next sought the origin of Ca^2+^ transients induced by GABA. The GABA‐induced Ca^2+^ transients were blocked by CdCl_2_ (100 *μ*mol/L), a non‐selective blocker of calcium channels (data not shown) suggesting that GABA_A_ receptor activation leads to voltage‐gated Ca^2+^ channel (VGCC) activation. We verified that GABA‐induced Ca^2+^ transients were abolished by the removal of extracellular Ca^2+^ (Fig. 1E, F) but not by the depletion of intracellular Ca^2+^ stores (Fig. 1G, H). We confirmed that Ca^2+^ transients induced by supramaximal GABA (300 *μ*mol/L) were blocked by the non‐competitive GABA_A_ receptor antagonist picrotoxin (Fig. 1I) in a concentration‐dependent manner (Fig. 1J). Ca^2+^ responses could also be induced by the GABA_A_ receptor selective agonist muscimol (10 *μ*mol/L), which were again blocked by picrotoxin (100 *μ*mol/L) (data not shown).

Gramacidin is an antibiotic agent which diffuses into the cell membrane forming small Cl^‐^ impermeable perforations, thereby giving electrical access without disruption of the intracellular Cl^‐^ concentration (Ebihara et al. 1995; Kyrozis and Reichling 1995). Using gramicidin‐perforated patch‐clamp technique we observed that GABA (300 *μ*mol/L) evoked a fast, rapidly decaying depolarization of the membrane potential in small‐sized (12–20 *μ*m diameter) DRG neurons (Fig. 1K). The average membrane potential was recorded as −53.7 ± 0.8 mV at rest and a peak of −35.3 ± 0.6 mV during GABA (300 *μ*mol/L) application (Fig. 1L); at this concentration of GABA action potentials were observed in 1/13 neurons (data not shown). Thus, GABA elicits membrane depolarization via activation of GABA_A_ receptors, resulting in VGCC‐mediated Ca^2+^ influx in DRG neurons.

### Peripheral GABA_A_ receptors are nociceptive in acute inflammation

**Figure 2:**
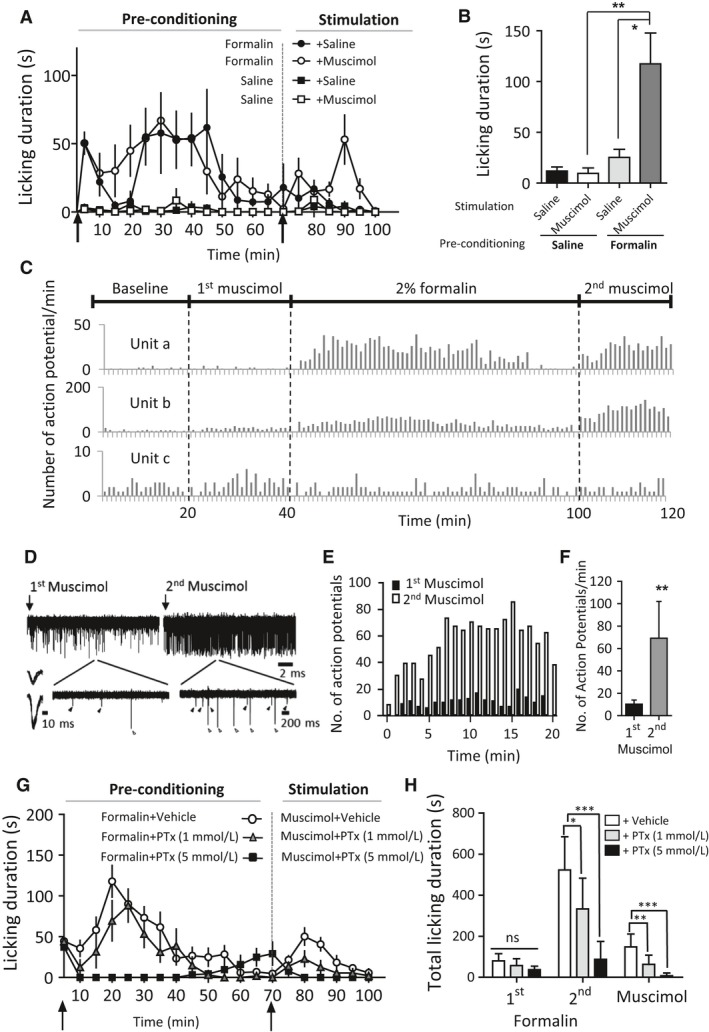
Activation of peripheral GABA_A_ receptors induces pain‐like behavior after acute formalin inflammation but not in naïve mice. (A) Time course of hind paw licking behaviors during pre‐conditioning with formalin (0.8%, 20 *μ*L) followed by injection of muscimol (1 mmol/L, 20 *μ*L) (open circles, *n* = 5 mice) or 0.9% saline (black circles, *n* = 4 mice) (‘stimulation’); pre‐conditioning with 0.9% saline vehicle followed by injection of muscimol (open squares, *n* = 5 mice) or 0.9% saline (black squares, *n* = 5 mice) into the same dorsum hind paw area. (B) GABA_A_ receptor agonist muscimol significantly increased hind paw licking behavior in formalin pre‐conditioning group. Bar graph represents accumulative licking time during 30 min stimulation phase (**P <* *0.01, one‐way ANOVA with Bonferroni post‐test compared with muscimol in saline pre‐conditioning group; (*P <* *0.05, one‐way ANOVA with Bonferroni post‐test compared with saline in formalin pre‐conditioning group). (C) Extracellular unit recording of spinal WDR neurons showing the pattern of action potential (AP) discharge frequency of three units (a, b, and c) in response to sequential injections of muscimol (1 mmol/L, 20 *μ*L) and formalin (0.8%, 20 *μ*L) into the hind paw. (D–F) Pre‐injection of formalin (0.8%) increases frequency of action potential (AP) discharge in spinal WDR neurons by injection of muscimol (1 mmol/L, 20 *μ*L) into the hind paw. (D) Representative traces showing the AP discharge of two units induced by muscimol before and after formalin injection. (E) Frequency histogram of muscimol‐induced AP discharge (1 min bins). (F) Mean frequency of total muscimol‐induced AP discharge per min for 20 min, *n* = 12 units recorded from 6 animals, **P <* *0.01, Wilcoxon signed‐rank test. (G) Effect of GABA_A_ antagonist picrotoxin (PTx) on time course of hind paw licking behaviors during pre‐conditioning with formalin (0.8%, 20 *μ*L) followed by injection of muscimol (1 mmol/L, 20 *μ*L) into the same dorsum hind paw area. Picrotoxin was given at 1 mmol/L (gray triangle, *n* = 5 mice), 5 mmol/L (black squares, *n* = 6 mice) or vehicle (0.9% saline, open circles, *n* = 12 mice). (H) Picrotoxin significantly inhibited the second phase of the formalin response (*P < 0.05, *** *P* < 0.001, one‐way ANOVA with Bonferroni post‐test) as well as muscimol‐induced pain behavior (**P < 0.01, *P* < 0.001, one‐way ANOVA with Bonferroni post‐test). WDR, wide dynamic range.

We investigated the modulatory effects of GABA_A_ receptors on inflammatory pain by injecting the GABA_A_ agonist muscimol (1 mmol/L, 20 *μ*L) into the hind paw after cessation of 0.8% formalin‐induced pain behavior in adult mice. This formalin pre‐conditioning protocol revealed a novel peripheral GABA_A_ receptor‐mediated nocifensive behavior (Fig. 2A, B). In contrast, mice injected with muscimol after saline pre‐injection showed no pain‐like behavior in the 30 min after muscimol injection (Fig. 2A, B). These results suggest that GABA_A_ receptors elicit nociceptive behavior in the presence of acute inflammation, but not in otherwise naïve animals.

We next investigated whether muscimol‐induced nociceptive signaling can be transmitted to the spinal cord after formalin inflammation by performing extracellular recording of WDR neurons in adult mice in vivo. After a period (20 min) of baseline recording, muscimol (1 mmol/L, 20 *μ*L) was injected subcutaneously into the hind paw. The spike frequency of all units did not differ compared with baseline in the 20 min after the first muscimol injection (Fig. 2C). Units representing the action potential discharge of 12 individual WDR neurons were identified based on spike profile (Fig 2D). Subsequent formalin (0.8%) injection augmented spike frequency in 8 out of 12 WDR neurons (Representative units ‘a’ and ‘b’, Fig. 2C). After the activity of formalin‐responsive neurons returned to baseline (approximately 60 min) a second muscimol injection facilitated action potential firing in 10 out of 12 WDR neurons. Units identified at a low frequency during the first muscimol injection were observed firing at higher frequency during the second muscimol injection after formalin conditioning (Fig. 2D, E). The overall number of action potentials recorded during the 20 min after the second muscimol injection was also significantly increased (Fig. 2F). Representative unit ‘c’, which did not respond to the formalin injection, was not affected by the second muscimol injection (Fig. 2C). These results indicate that activation of GABA_A_ receptors in peripheral nociceptive neurons after acute inflammation evokes nociceptive signal transmission to second‐order neurons in the spinal cord. In addition, we confirmed that muscimol‐induced licking behavior after formalin pre‐conditioning was dose‐dependently inhibited by the non‐competitive GABA_A_ receptor antagonist picrotoxin (Fig. 2G, H). Interestingly, co‐injection of picrotoxin with formalin also dose‐dependently inhibited the second phase of the formalin‐induced behavior.

### Formalin potentiates GABA response independent of TRPA1

**Figure 3:**
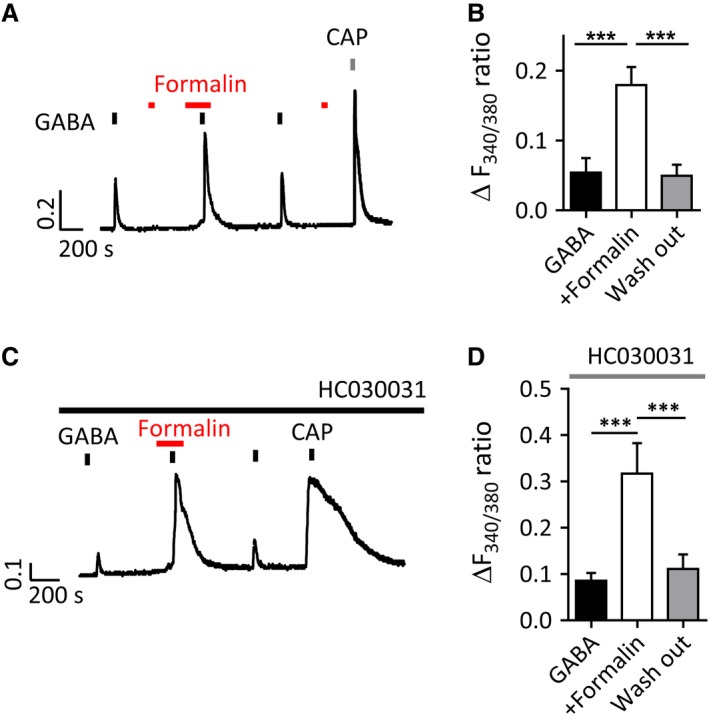
Formalin potentiates GABA‐induced Ca^2+^ transients in a TRPA1 independent manner. (A, B) Formalin potentiates GABA‐induced Ca^2+^ transients in nociceptive neurons. (A) Representative traces showing potentiated GABA (300 *μ*mol/L)‐induced Ca^2+^ by formalin pre‐treatment (0.001%, 120 sec) in a capsaicin‐sensitive DRG neuron. (B) Mean amplitude of GABA‐induced Ca^2+^ transients (*n* = 18 cells, 6 coverslips from 3 mice; ***P <* *0.001, one‐way ANOVA). (C) Potentiated GABA‐induced Ca^2+^ transients by formalin (0.001%) are not blocked by TRPA1 receptor selective antagonist HC030031 (30 *μ*mol/L). (D) Quantification of GABA‐induced Ca^2+^ transients in capsaicin‐sensitive DRG neurons (*n* = 16 cells, 3 coverslips from 2 mice; ***P <* *0.001, one‐way ANOVA with Bonferroni post‐test). DRG, dorsal root ganglia.

We first examined the effect of formalin on GABA‐induced Ca^2+^ transients in nociceptive DRG neurons. GABA‐induced Ca^2+^ transient amplitudes were increased after pre‐treatment of formalin (0.001%, 2 min) (Fig. 3A, B). Formalin is known to directly activate nociceptive neurons through TRPA1 (McNamara et al. 2007); however, formalin potentiation of GABA‐induced Ca^2+^ transients occurred in neurons which had no response to formalin (0.001%) alone (Fig. 3A) and furthermore was not affected by the TRPA1 selective antagonist, HC030031 (30 *μ*mol/L) (Fig. 3C, D).

### Prostaglandin E2 reveals GABA_A_ receptor‐mediated nociception

**Figure 4:**
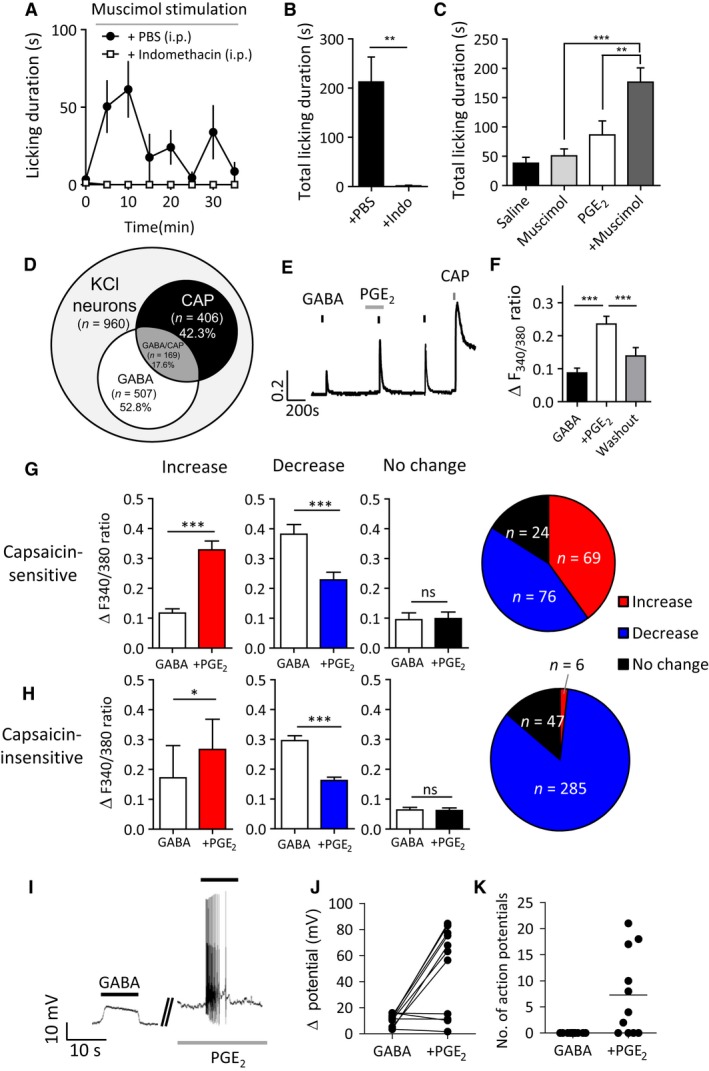
Prostaglandin E2 contributes to GABA_A_ receptor‐mediated pain and neuronal excitability. (A) Effect of intraperitoneal injection of indomethacin (40 mg/kg) (open squares) or PBS vehicle (black circles) on muscimol (1 mmol/L, 20 *μ*L)‐induced hind paw licking behavior in formalin (0.8%, 20 *μ*L) preconditioned mice. (B) Cyclooxygenase inhibitor indomethacin abolishes muscimol‐induced hind paw licking behavior after formalin pre‐conditioning (*n* = 6 mice per group, **P <* *0.05, Student's *t*‐test). (C) PGE_2_ potentiates muscimol‐induced hind paw licking behavior. Quantification represents total duration of hind paw licking behavior during 30 min after PGE_2_ (10 nmol, 20 *μ*L) or saline vehicle with or without muscimol (1 mmol/L, 20 *μ*L) (*n* = 5–6 mice per group, *P < 0.05, ***P <* *0.001, one‐way ANOVA with Bonferroni post‐test). (D) Venn diagram of GABA (300 *μ*mol/L) and capsaicin (CAP; 1 *μ*mol/L) sensitivity among KCl‐responsive neuronal cells in acute (<24 h) cultures of adult mouse DRG (*n* = 960 cells, 19 coverslips from 3 mice). (E, F) PGE_2_ potentiates GABA‐induced Ca^2+^ transients in nociceptive neurons. (E) Representative trace showing potentiated GABA (300 *μ*mol/L)‐induced Ca^2+^ by PGE_2_ pre‐treatment (10 *μ*mol/L, 180 sec) in a capsaicin‐sensitive DRG neuron. (F) Mean amplitude of GABA‐induced GABA‐induced Ca^2+^ transients (*n* = 34 cells, 11 coverslips from 7 mice; ***P <* *0.001, one‐way ANOVA). (G, H) The effect of PGE_2_ on the amplitude of GABA‐induced Ca^2+^ transients. GABA‐induced Ca^2+^ transients in capsaicin‐sensitive (G) and capsaicin‐insensitive (H) GABA‐responsive neurons were classified according to whether the amplitude (change in F_340/380_ ratio) was increased >0.03 (‘potentiated’), decreased >0.02, or unchanged (−0.02 to +0.03) during PGE_2_ (10 *μ*mol/L) compared to control responses (*P < 0.05, ***P < 0.001, paired two‐tailed t‐test). (I, J, K) PGE_2_ increases GABA‐induced neuronal excitability. (I) Representative traces of GABA (100 *μ*mol/L)‐induced depolarization before and after pre‐incubation with PGE_2_ (10 *μ*mol/L, 180 sec). (J) Quantification of GABA‐induced changes in membrane potentials and (K) number of action potentials by GABA before and after pre‐incubation with PGE_2_ (10 *μ*mol/L) (*n* = 21 cells). DRG, dorsal root ganglia.

We next sought to determine the mechanism underlying peripheral GABA_A_ receptor‐induced nociception in inflammation. Formalin‐induced pain behavior can be attenuated by pre‐treatment with the cyclooxygenase (COX) inhibitor indomethacin (Hunskaar et al. 1986), which reduces inflammation by blocking prostanoid synthesis. We found that systemic pre‐treatment with indomethacin (40 mg/kg, i.p.) 30 min before formalin injection completely abolished the subsequent muscimol‐induced licking behavior in adult mice (Fig. 4A, B). The prostaglandin E2 (PGE_2_) derivative is a potent inflammatory mediator produced at the site of inflammation (Fulton et al. 2006). We observed that PGE_2_ (500 *μ*mol/L, 20 *μ*L)‐induced licking behavior was also enhanced by co‐injection of muscimol (Fig. 4C), confirming that PGE_2_ is sufficient for GABA_A_ receptor‐mediated nocifensive behavior.

Next, we examined the effect of PGE_2_ on GABA‐induced Ca^2+^ transients in cultured DRG neurons. Nociceptive DRG neurons were identified by their response to a 10 sec application of capsaicin (1 *μ*mol/L) at the end of each experiment and made up 42.3% (406/960 neurons) of the total population, of which 17.6% (169/960 neurons) were also responsive to GABA (Fig. 4D). We found that PGE_2_ (10 *μ*mol/L) potentiated GABA‐induced Ca^2+^ transients (Fig. 4E, F) in a large subpopulation of capsaicin‐responsive nociceptive DRG neurons (40.1%; *n* = 69/169 neurons) (Fig. 4G) but only a small number of capsaicin‐insensitive DRG (1.8%; *n* = 6/338 neurons) (Fig. 4H). In addition, PGE_2_ (10 *μ*mol/L) facilitated GABA‐induced membrane depolarization resulting in action potential firing in a subpopulation of small‐sized DRG neurons as recorded by gramicidin perforated patch (Fig. 4I–K). Collectively, our results suggest that PGE_2_ is one of the possible pro‐inflammatory mediators that may contribute to GABA_A_ receptor‐mediated nocifensive behavior during acute inflammation via action in a subpopulation of nociceptive DRG neurons.

### Activation of EP4 receptors increases GABA‐induced nociceptive neuron activity

**Figure 5:**
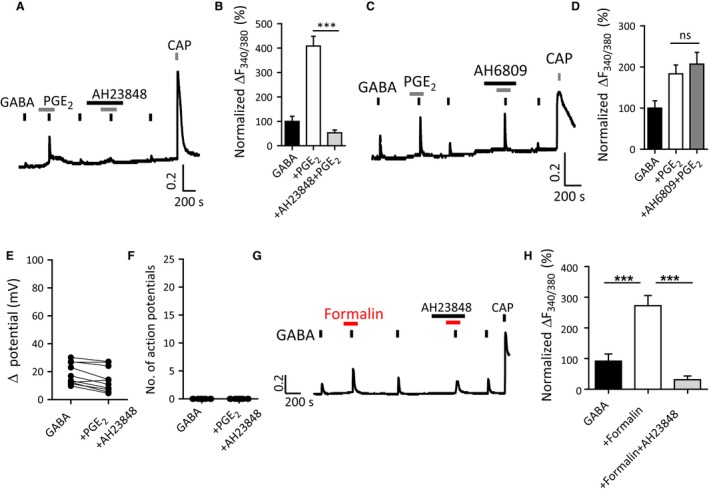
Potentiation of GABA‐induced neuronal excitability is mediated by EP4 receptors. (A) Potentiation of GABA (300 *μ*mol/L)‐induced Ca^2+^ transients in capsaicin‐sensitive DRG neurons by PGE_2_ (10 *μ*mol/L) is inhibited by the EP4 receptor antagonist AH23848 (10 *μ*mol/L) (B) Quantification of GABA‐induced Ca^2+^ transients relative to peak amplitude of 1st GABA response in the presence of AH23848 (*n* = 19 cells, 12 coverslips from 4 mice; ***P <* *0.001, one‐way ANOVA with Bonferroni post‐test) (C) Potentiation of GABA (300 *μ*mol/L)‐induced Ca^2+^ transients by PGE_2_ (10 *μ*mol/L) is not affected by EP1‐2 receptor antagonist AH6809 (50 *μ*mol/L). (D) Quantification of GABA‐induced Ca^2+^ transients relative to peak amplitude of 1st GABA response in the presence of AH6809 (*n* = 15 cells, 5 coverslips from 3 mice). (E, F) The potentiating effect of PGE_2_ on GABA‐induced changes in membrane potentials (E) and generation of action potentials (F) is abolished by EP4 receptor antagonist AH23848 (10 *μ*mol/L) in small‐sized DRG neurons (*n* = 10 cells). (G) Potentiated GABA (300 *μ*mol/L)‐induced Ca^2+^ transients by formalin (0.001%) is blocked by EP4 receptor antagonist AH23848 (10 *μ*mol/L) in capsaicin‐sensitive DRG neurons. (H) Quantification of potentiated GABA‐induced Ca^2+^ transients by formalin (0.001%) in the presence of AH23848 relative to peak amplitude of first GABA response (*n* = 8 cells, 6 coverslips from 3 mice; ***P <* *0.001, repeat‐measures one‐way ANOVA with Bonferroni post‐test). DRG, dorsal root ganglia.

PGE_2_ signals through a family of EP receptors (EP1‐4) of which subtypes EP1, EP2, and EP4 are excitatory and promote inflammation (Fulton et al. 2006). PGE_2_‐potentiated GABA responses were inhibited by EP4 receptor antagonist, AH23848 (10 *μ*mol/L) (Lin et al. 2006) (Fig 5A, B) but not by EP1‐2 receptor antagonist, AH6809 (50 *μ*mol/L) (St‐Jacques and Ma 2011) (Fig. 5C, D). GABA‐induced action potential firing in the presence of PGE_2_ was also abolished by AH23848 (Fig. 5E, F). Furthermore, potentiation of GABA‐induced Ca^2+^ transients by formalin was also blocked by AH23848 (10 *μ*mol/L) (Fig. 5G, H). Together, these data suggest that formalin and PGE_2_ may potentiate GABA‐induced responses through an EP4 receptor signaling mechanism in nociceptive DRG neurons.

### Voltage‐gated Na^+^ channels may contribute to GABA‐induced neuronal excitability during PGE_2_ sensitization

PGE_2_ is an important mediator in the development of peripheral inflammation and peripheral sensitization (Vane 1971; Julius and Basbaum 2001). For example, PGE_2_ can directly potentiate the TTX‐resistant Na^+^ channels Na_v_1.8 (England et al. 1996; Gold et al. 1998) and Na_v_1.9 (Rush and Waxman 2004) in peripheral nociceptive neurons. We therefore conducted experiments to examine whether modulation of Na_v_ channels contributes to GABA‐induced responses during PGE_2_ application.

**Figure 6:**
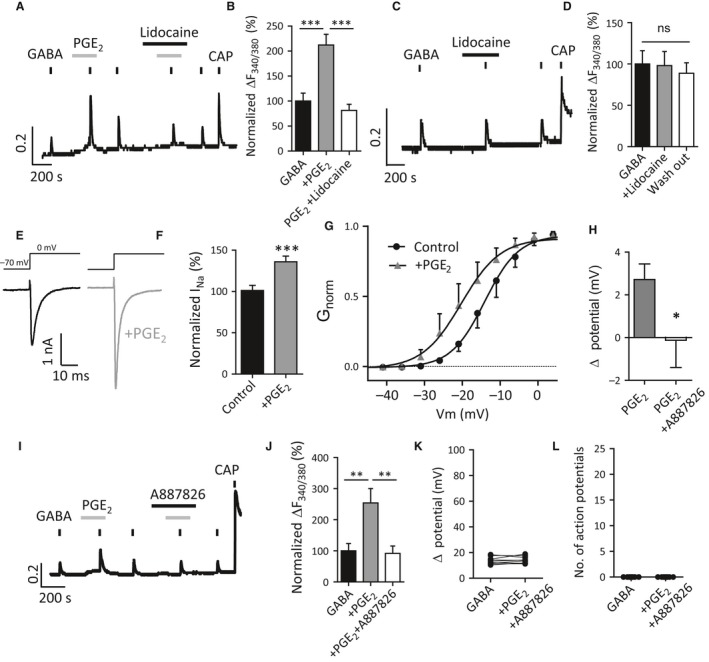
PGE_2_ potentiates GABA –induced neuronal excitability through Na_v_1.8 channel modulation. (A) Potentiation of GABA (300 *μ*mol/L)‐induced Ca^2+^ transients by PGE_2_ (10 *μ*mol/L) is inhibited by voltage‐gated sodium channel blocker lidocaine (300 *μ*mol/L) in capsaicin‐sensitive DRG neurons. (B) Quantification of GABA‐induced Ca^2+^ transients in the presence and absence of lidocaine relative to peak amplitude of 1st GABA response (*n* = 12 cells, 9 coverslips from 4 mice; ***P <* *0.001, repeat‐measures one‐way ANOVA with Bonferroni post‐test). (C) GABA‐induced Ca^2+^ transients are unaffected by lidocaine (300 *μ*mol/L, 360 sec). (D) Quantification of GABA‐induced Ca^2+^ transients amplitudes in the presence of lidocaine (*n* = 17 cells, 4 coverslips from 3 mice; one‐way ANOVA with Bonferroni post‐test). (E) PGE_2_ (10 *μ*mol/L, 180 sec) potentiates TTX‐resistant sodium currents in small‐sized DRG neurons. (F) Quantification of normalized TTX‐resistant sodium currents amplitude (*n* = 5 cells, ***P <* *0.001, paired Student's *t*‐test). (G) Normalized conductance‐voltage relationship of Na_v_1.8 currents in the presence and absence of PGE_2_ (10 *μ*mol/L) (*n* = 6 cells) fit with a Boltzmann function. Test pulses were preceded by a 500‐msec step to −50 mV to inactivate TTX‐resistant Na_v_1.9. (H) A887826 (100 nmol/L) inhibits the membrane depolarization caused by PGE_2_ (10 *μ*mol/L) application (*n* = 12–20 cells; *P <* *0.05, unpaired Student's *t*‐test). (I) Potentiation of GABA (300 *μ*mol/L)‐induced Ca^2+^ transients by PGE_2_ (10 *μ*mol/L) is abolished by Na_v_1.8 channel blocker A887826 (100 nmol/L) in DRG neurons (J) Quantification of GABA‐induced Ca^2+^ transients in the presence and absence of A887826 relative to peak amplitude of 1st GABA response (*n* = 10 cells, 4 coverslips from 3 mice; **P <* *0.01, repeat‐ measures one‐way ANOVA with Bonferroni post‐test). (K, L) The potentiating effect of PGE_2_ on GABA‐induced changes in membrane potentials (K) and generating action potentials (L) in small‐sized DRG neurons is blocked by Na_v_1.8 channel blocker A887826 (100 nmol/L) (*n* = 9 cells). DRG, dorsal root ganglia.

The archetypal Na_v_ channel blocker lidocaine (Sheets et al. 2008) was applied to cultured DRG neurons during PGE_2_‐induced potentiation of the GABA response (Fig. 6A). Lidocaine (300 *μ*mol/L) inhibited the potentiation of GABA‐induced Ca^2+^ responses by PGE_2_ (Fig. 6A, B) but had no effect on GABA‐induced Ca^2+^ transients in control conditions (Fig. 6C, D). We confirmed that PGE_2_ acutely potentiates TTX‐resistant Na_v_ channel current (Fig 6E, F), as well as produce a leftward shift (−6.08 ± 1.8 mV, *n* = 6 cells) in the voltage dependence of Na_v_1.8 activation (Fig 6G). PGE_2_ additionally induced a small depolarization from the resting membrane potential (Fig. 6H). Pre‐incubation with the Na_v_1.8 channel antagonist A887826 (100 nmol/L) (Zhang et al. 2010) not only blocked PGE_2_‐induced depolarization (Fig. 6H) but also the PGE_2_ potentiation of GABA‐induced Ca^2+^ responses (Fig. 6I, J) as well as GABA‐induced action potential firing in the presence of PGE_2_ (Fig. 6K, L). Together, these results suggest that the activation of voltage‐sensitive TTX‐resistant Na^+^ channels may contribute to the nociceptive role of GABA_A_ receptors during PGE_2_‐mediated inflammation.

### NKCC1 is not required for potentiated GABA responses by pro‐inflammatory mediators

**Figure 7:**
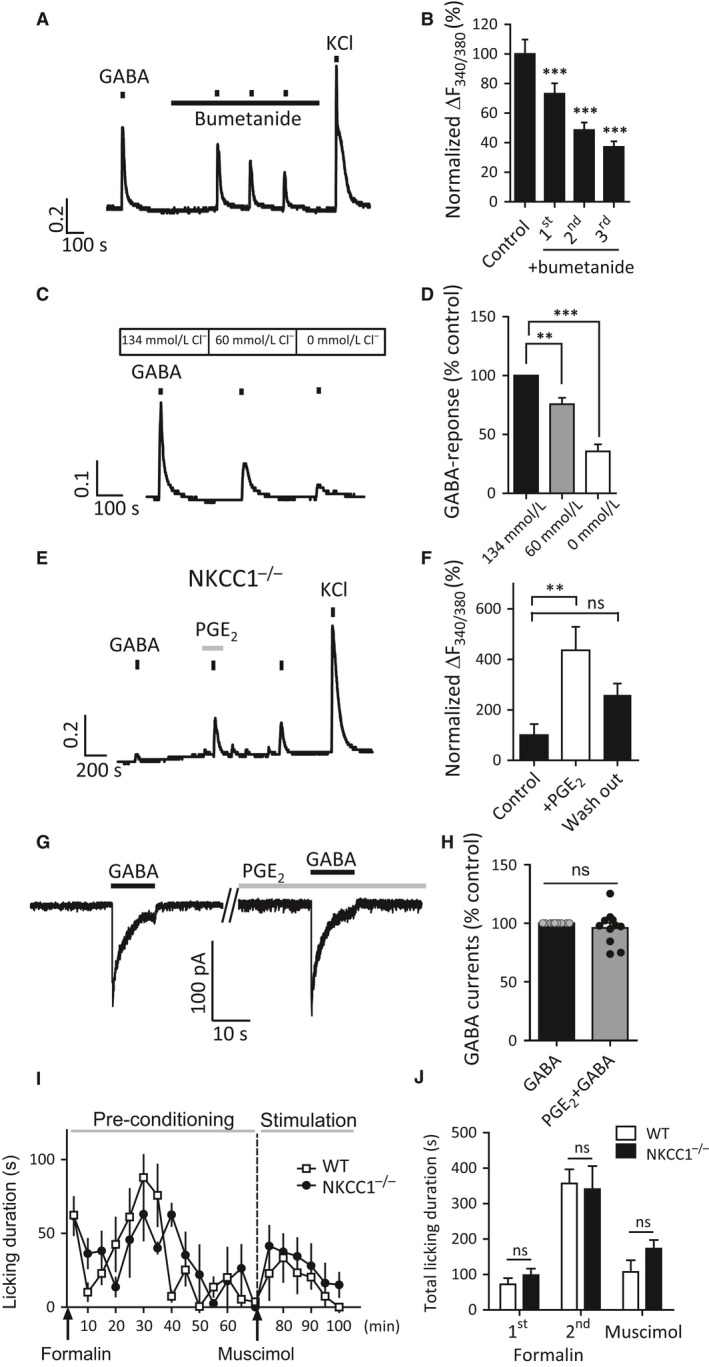
NKCC1 is not required for GABA_A_ receptor‐mediated pain behavior in formalin inflammation. (A) GABA‐induced Ca^2+^ transients are decreased by sequential application of GABA (300 *μ*mol/L) in the presence of NKCC1 co‐transporter inhibitor bumetanide (10 *μ*mol/L). (B) Quantification of GABA‐induced Ca^2+^ transients normalized by 1st GABA response (*n* = 24 cells, 6 coverslips from 3 mice; **P <* *0.01, ***P <* *0.001, one‐way ANOVA with Bonferroni post‐test. (C) GABA (300 *μ*mol/L)‐induced Ca^2+^ transient amplitudes vary with different concentrations of extracellular Cl^−^ in dissociated DRG neurons. (D) Quantification of GABA‐induced Ca^2+^ transients normalized by the amplitude of Ca^2+^ transients in control extracellular solution with 134 mmol/L Cl^−^ (*n* = 10 cells, 5 coverslips from 4 mice; **P <* *0.01, ***P <* *0.001, repeat‐measures ANOVA with Bonferroni post‐test). (E) Representative traces showing potentiation of GABA (300 *μ*mol/L)‐induced Ca^2+^ transients by PGE_2_ pre‐treatment (10 *μ*mol/L, 180 sec) in NKCC1^−/−^ DRG neurons. (F) Normalized amplitude of GABA‐induced Ca^2+^ transients in NKCC1^−/−^ DRG neurons (*n* = 12 cells, 4 coverslips from 3 mice; **P <* *0.01, repeat‐measures one‐way ANOVA with Bonferroni post‐test). (G, H) PGE_2_ (10 *μ*mol/L) does not affect GABA (100 *μ*mol/L)‐induced currents at a holding potential of −60 mV in small‐size DRG neurons (*n* = 11 cells, two‐tailed paired Student's *t*‐test). (I, J) Formalin‐evoked pain behavior and GABA_A_ receptor agonist muscimol‐induced pain behavior after formalin test are not altered in NKCC1^−/−^ mice. Formalin (0.8%, 20 *μ*L)‐induced biphasic licking behavior (1st and 2nd phases) followed by the injection of muscimol (1 mmol/L, 20 *μ*L) (GABA_A_ receptor‐mediated pain) in wild type (open squares) and NKCC1^−/−^ (black circles) mice. (F) Bar graph represents accumulative licking time during 1st (0–10 min) and 2nd phases (10–70 min) of formalin‐induced pain‐like behavior and muscimol‐induced pain‐like behavior (70–100 min) (*n* = 5 mice per genotype; two‐way ANOVA compared with WT mice). DRG, dorsal root ganglia.

The Na^+^‐K^+^‐Cl^‐^‐co‐transporter (NKCC1) is thought to be responsible for maintaining intracellular Cl^‐^ levels in DRG neurons (Sung et al. 2000). Furthermore, NKCC1 activity is dynamically regulated by cAMP‐dependent protein kinase (PKA) and protein kinase C (PKC) phosphorylation (Smith et al. 2008; Flemmer et al. 2010). We therefore hypothesized that NKCC1 may be a downstream target of EP4 receptor signaling (via PKA) during formalin‐induced inflammation leading to potentiation of GABA_A_ receptor‐mediated responses. To investigate whether NKCC1 activity is required for GABA‐induced Ca^2+^ transients, we applied the NKCC1 inhibitor bumetanide. Continuous application of bumetanide (10 *μ*mol/L) led to a progressive reduction in amplitude of GABA‐induced Ca^2+^ transients (Fig. 7A, B). To confirm the Cl^−^ dependency of GABA‐induced Ca^2+^ transients extracellular Cl^−^ was substituted with gluconate; this has the effect of gradually depleting intracellular Cl^−^ and effectively reducing the Cl^−^ concentration gradient (Rocha‐Gonzalez et al. 2008). Under such conditions, we saw a consistent reduction in GABA‐induced Ca^2+^ transient amplitude (Fig. 7C, D). Reduction of extracellular Cl^−^ in the bath solution to 0 mmol/L over just 15 min reduced GABA‐induced Ca^2+^ transient amplitude by more than 60% (Fig. 7D). However, potentiated GABA‐induced Ca^2+^ transients by PGE_2_ was still observed in DRG neurons isolated from NKCC1‐deficient mice (Fig. 7E, F). In addition, application of PGE_2_ for the duration necessary to potentiate GABA‐induced Ca^2+^ transients did not significantly alter the amplitude of GABA‐induced currents (Fig. 7G, H). Furthermore, peripheral injection of muscimol still restored licking behavior after formalin pre‐conditioning in mice lacking NKCC1 (Fig. 7I, J). These results suggest that NKCC1 activity is not required for peripheral GABA_A_‐mediated nociception during acute inflammation.

## Discussion

### Peripheral GABA_A_ receptor nociception in vivo

GABA has long been observed to depolarize peripheral sensory nerves in rodents (Feltz and Rasminsky 1974; Deschenes et al. 1976; Sung et al. 2000) and humans (Carr et al. 2010); however, activation of peripheral GABA_A_ receptors elicits neither spontaneous pain behavior nor mechanical sensitivity in naïve animals. One proposal to this apparent paradox is the phenomenon of primary afferent depolarization (Rudomin and Schmidt 1999; Willis 1999) whereby sub‐threshold depolarization results in inactivation of voltage‐gated channels. Nevertheless, previous reports have suggested that the activation of peripheral GABA_A_ receptors can facilitate inflammation‐induced pain behavior (Carlton et al. 1999; Bravo‐Hernandez et al. 2014). The formalin test is a well‐characterized test of acute‐inflammatory pain (Hunskaar and Hole 1987) which produces biphasic pain‐like behavior that disappears 60 min after the original treatment. By a simple modification of the protocol ‐ injection of muscimol *after* the return of formalin behavior to baseline ‐ we were able to reveal a novel GABA_A_ receptor‐mediated nocifensive behavior that could be used for further mechanistic study.

The phenomenon of GABA_A_‐mediated nociception after acute inflammation appears to contrast with observations that either application of GABA_A_ agonists directly to the DRG (Naik et al. 2008), or systemic administration of GABA_A_ allosteric modulators (Munro et al. 2008), can have an alleviating effect in certain models of chronic inflammatory or neuropathic pain, although the contribution of spinal GABA_A_ receptors cannot be completely excluded in these studies.

### Ca^2+^ imaging as an indirect measure of GABA_A_ receptor function in vitro

Unlike whole‐cell techniques, the fura2‐AM Ca^2+^ imaging technique allows the quantitative study of cell activity without disrupting the cell membrane. Thus, cells may be studied in a relatively undisturbed state with endogenous concentrations of intracellular ions and signaling factors. Application of GABA to DRG neurons induced consistent and concentration‐dependent Ca^2+^ transients that were mediated by GABA_A_ receptors specifically and blocked by CdCl_2_, a non‐selective voltage‐gated Ca^2+^ channel blocker. Moreover, manipulation of the Cl^‐^ concentration gradient had a proportional effect on GABA‐induced Ca^2+^ transient amplitude. These results suggested that GABA elicited Ca^2+^ influx through VGCC activation in DRG neurons of an amplitude proportional to the degree of membrane depolarization.

The high concentration of muscimol (>1 mmol/L) required to potentiate the formalin nociceptive response in vivo in this study and reported by others (Carlton et al. 1999) suggests that near‐maximal activation of GABA_A_ receptor is required for conversion to a nociceptive role. Correspondingly, we used a relatively high concentration of GABA (300 *μ*mol/L) for the majority of our in vitro studies, which represented a supramaximal response in our Ca^2+^ assay. The physiologic concentration of GABA has been reported at >500 *μ*mol/L at GABAergic synapses in the brain (Maconochie et al. 1994) with a peak concentration of 1.5–3 mmol/L GABA (Mozrzymas et al. 2003). Further work will be required to establish whether such concentrations are reached at peripheral GABA_A_ receptors in vivo.

### Formalin and PGE_2_ potentiation of GABA responses are mediated by EP4 receptor

The effect of formalin on DRG neurons in our in vitro culture system appeared to mimic the in vivo observation by potentiating the GABA‐induced Ca^2+^ response. Formalin‐induced pain behavior is characterized by two phases: an acute first phase mediated by peripheral nociceptive transmission and a second phase thought to be driven by a combination of central sensitization (McNamara et al. 2007) and biphasic nociceptor activity (McCall et al. 1996). In addition, formalin is known to directly activate TRPA1 in nociceptive sensory neurons. However, a supramaximal concentration of the TRPA1 selective antagonist, HC030031, had no effect on the potentiation of the GABA‐induced Ca^2+^ responses by acute low concentration (0.001%) formalin suggesting a mechanism independent of TRPA1.

PGE_2_ is a potent inflammatory mediator produced during formalin‐induced inflammation (Malmberg and Yaksh 1995). PGE_2_ sensitizes peripheral nociceptive neurons through EP receptors present on the peripheral terminals of high‐threshold sensory neurons (Omote et al. 2002). In our experiments, PGE_2_ potentiated GABA‐induced Ca^2+^ transients almost exclusively in capsaicin‐sensitive DRG neurons suggesting that the effect is restricted to a population of nociceptive sensory neurons. PGE_2_ also revealed additional muscimol‐induced pain‐like licking behaviors in vivo.

There are four subtypes of PGE_2_ receptor known as EP receptors (EP1‐4), all of which are G‐protein coupled receptors: EP1 coupled to G_q_/G11, EP2 and EP4 coupled to G_s_, and EP3 coupled to G_s_ and G_i_. In particular, the EP4 receptor is highly expressed in primary sensory neurons and EP4 levels are known to increase in the DRG after peripheral inflammation (Lin et al. 2006). We found that GABA‐induced Ca^2+^ transients potentiated by PGE_2_ were blocked by the EP4 receptor antagonist, AH23848, but not by EP1‐2 receptor antagonist, AH6809. Furthermore, formalin‐induced facilitation of GABA‐induced Ca^2+^ transients was also abolished by AH23848. These observations suggest that potentiation of the GABA response by formalin and PGE_2_ may share the same downstream pathway through the EP4 receptor, although in the case of formalin we cannot exclude the possible contribution from other inflammatory mediators. Formalin application to DRG neurons may act indirectly on EP4 receptors via PGE_2_ release. The source of PGE_2_ released in our DRG cultures by formalin application in vitro is currently unknown but could include non‐neuronal cells carried over during DRG dissociation and plating.

### Sensitization of Na_v_ channels may increase the gain of GABA‐induced responses in DRG

Previous studies have shown that the PGE_2_ signaling pathway can modulate TTX‐resistant Na^+^ channels to sensitize peripheral sensory neurons (England et al. 1996). We found that lidocaine blocked PGE_2_‐induced potentiation of GABA‐induced Ca^2+^ transients, but did not affect the basal GABA‐induced Ca^2+^ response, suggesting that simultaneous Na^+^ and Cl^−^ conductance are necessary for the conversion to a nociceptive state. To confirm the molecular link between PGE_2_‐induced sensitization and GABA signaling in primary sensory neurons, we applied the TTX‐resistant Na_v_1.8 channel blocker A887826 during the PGE_2_ treatment, which successfully prevented the potentiation of GABA signaling by PGE_2_. In some recordings (for example Fig. 4E), the potentiating effect of PGE_2_ on GABA‐induced Ca^2+^‐transients appeared to last after washout until the following GABA application. However, on average GABA‐induced Ca^2+^ transients returned to near their pre‐PGE_2_ control amplitude (Fig. 4F). This is consistent with the earlier work on PGE_2_ sensitization of TTX‐resistant Na^+^ channels where currents returned to their pre‐exposure amplitudes within a few minutes of PGE2 washout (Gold et al. 1996).

Our perforated patch‐clamp experiments show that sub‐threshold GABA‐induced depolarizations in control conditions were enhanced during PGE_2_ treatment and commonly resulted in action potentials. It has long been known that PGE_2_ causes a leftward shift in the activation curve of TTX‐resistant Na^+^ currents (England et al. 1996; Gold et al. 1996). In small‐sized DRG neurons GABA_A_ receptor activation depolarized the membrane potential to approximately −35 mV (Fig. 1N), which just lies within the activation range of PGE_2_‐sensitized Na_v_1.8 (See Fig. 6G); we speculate that this may lead ultimately to the action potential firing that we observe during application of GABA in the presence of PGE_2_. These effects were completely prevented by selective EP4 receptor and Na_v_1.8 channel antagonists, corroborating the pharmacology suggested by our Ca^2+^ imaging results. The concentration of A887826 used in this study (100 nmol/L) is consistent with the selective blockade of Na_v_1.8 in a heterologous expression system as well as rodent DRG (Zhang et al. 2010), although the contribution from the TTX‐resistant channel Na_v_1.9, which is also sensitized by PGE_2_ (Rush and Waxman 2004), cannot be ruled out. Further work however will be needed to elucidate the relationship between PGE_2_‐induced sensitization of voltage‐gated Na^+^ channels and GABA_A_ receptor‐mediated depolarization in vivo.

An increase in the responsiveness of isolated DRG neurons to GABA has previously been observed with Ca^2+^ imaging after chronic (3 days complete Freund's adjuvant) inflammation in adult rats, and occurs concurrent with a decrease in low threshold K^+^ current density leading to membrane depolarization (Zhu et al. 2012b). Interestingly, this and another study by the same authors (Zhu et al. 2012a) report an increase in GABA current density with chronic inflammation, something that we did not observe in the presence of acute PGE_2_ application. Notwithstanding the differences in acute and chronic inflammation we cannot therefore exclude the contribution of other ion conductances in the mechanism(s) of GABA‐mediated nociception.

### NKCC1 is not required for GABA_A_ nociception in inflammation

As we show in our in vitro characterization, GABA induces cell membrane depolarization in peripheral sensory neurons because of the high intracellular Cl^−^ concentration of DRG neurons. We further confirm that the GABA response in isolated DRG neurons is maintained by the constitutive activity of the Na^+^‐K^+^‐2Cl^‐^ co‐transporter NKCC1. A previous study reported that intracellular Cl^−^ concentration was increased in DRG neurons 1 h after treatment with a soup of inflammatory mediators (Funk et al. 2008). This has led to the suggestion that pathological upregulation of NKCC1 in inflammation could initiate the transition of peripheral GABA_A_ receptors to a nociceptive role (Morales‐Aza et al. 2004). NKCC1 also represents a potential downstream target of EP4 signaling via PKA‐mediated phosphorylation (Flemmer et al. 2010). Despite the evidence in favor of a role for NKCC1 in GABA_A_‐mediated nociception, our data surprisingly show the maintenance of muscimol‐induced spontaneous licking behavior after formalin in mice lacking NKCC1, as well as PGE_2_‐induced potentiation of GABA‐induced Ca^2+^ transients in NKCC1^−/−^ DRG neurons. Although intracellular Cl^−^ is reduced in the DRG of NKCC1^−/−^ mice it is not completely eliminated (Sung et al. 2000), suggesting that compensation by another Cl^−^ regulatory mechanism allows depolarization by GABA in these mice. Our results also show that an acute application of PGE_2_ is sufficient to potentiate the GABA response, although we cannot exclude the possibility of an effect on intracellular Cl^−^ concentration via NKCC1 modulation with more chronic exposure to PGE_2_.

### Endogenous peripheral GABA_A_ receptor activation

As well as blocking muscimol‐induced behavior, high dose picrotoxin in the hind paw also inhibited the second phase of the formalin response. This surprising result is consistent with a previous study in which systemic picrotoxin treatment reduced formalin behavior (Heidari et al. 1996) and suggests that endogenous activators of the GABA_A_ receptor may indeed play a role in the behavioral response to acute formalin inflammation.

One important remaining question is the endogenous source of GABA_A_ receptor activation in DRG neurons. Expression of functional GABA_A_ receptors at the central terminals of DRG (Labrakakis et al. 2003) creates the potential to receive spinal GABAergic signaling. Expression of GABA, or the GABA‐synthesizing enzyme glutamine decarboxylase (GAD), has also been reported in various peripheral tissues, including keratinocytes (Ito et al. 2007) and macrophage (Tannahill et al. 2013). Whether these tissues are capable of synthesizing and more importantly releasing GABA remains to be explored.

In conclusion, our report suggests a nociceptive role of peripheral GABA_A_ receptors in acute‐inflammatory pain and presents a working model of the mechanism of GABA_A_ receptor‐mediated nociception.

## Conflict of Interests

All the authors have no competing interest in this study.
